# Adaptive Skid-Steering Control Approach for Robots on Uncertain Inclined Planes with Redundant Load-Bearing Mobility

**DOI:** 10.3390/biomimetics9020064

**Published:** 2024-01-23

**Authors:** Lin Zhang, Baoyu Wang, Enguang Guan, Xun Liu, Muhammad Saqib, Yanzheng Zhao

**Affiliations:** 1 School of Mechanical Engineering, Shanghai Jiao Tong University, Shanghai 200240, China; linzhang-sjtu@sjtu.edu.cn (L.Z.); liux_robot@sjtu.edu.cn (X.L.); 2 School of Mechanical Engineering, Zhejiang Sci-Tech University, Hangzhou 310018, China; wangbaoyu@drivedream.com; 3 Logistics Engineering College, Shanghai Maritime University, Shanghai 201306, China; egguan@shmtu.edu.cn; 4 Drivedream Machinery Equipment Co., Ltd., Shanghai 200240, China; muhammadsaqib@drivedream.com

**Keywords:** climbing manipulation, skid-steering, adaptive control, mobile robotics

## Abstract

Climbing manufacturing robots can create a revolutionary manufacturing paradigm for large and complex components, while the motion control of climbing manipulation-oriented robots (CMo-Rs) is still challenging considering anti-slippage problems. In this study, a CMo-R with full-scenery climbing capability and redundant load-bearing mobility is designed based on magnetic adsorption. A four-wheel kinematic model considering the slipping phenomenon is established. An adaptive kinematic control algorithm based on slip estimation using Lyapunov theory is designed for uncertain inclined planes. For comparison, the traditional PID-based algorithm without slip consideration is implemented as well. Numeric simulations are conducted to tackle the trajectory tracking problems for both circular and linear trajectories on the horizontal plane (HP), 50° inclined plane (50° IP), 60° inclined plane (60° IP), and vertical plane (VP). The results prove that our approach achieves better tracking accuracy. It demonstrated applicability in various climbing scenarios with uncertain inclined planes. The results of experiments also validate the feasibility, applicability, and stability of the proposed approach.

## 1. Introduction

Robotized intelligent manipulation is a growing trend in the manufacturing of large and complex components in aviation, aerospace, marine engineering, and even bionic applications [1]. Climbing manufacturing robots can create a revolutionary manufacturing paradigm for large and complex components [2]. Climbing robots have been successfully used for inspection [3], maintenance [4], and manufacturing tasks [5] in various fields. For climbing manipulation, the movability of the platform is one of the most important requirements. To move flexibly on the surface to be manufactured according to craft requirements, the technologies, e.g., adhesion mechanism and skid-steering control, should be reviewed first.

Regarding the adhesion technologies of climbing robots, researchers have conducted extensive studies and achieved significant results [6]. Based on different adhesion methods, existing climbing robots can mainly be classified as having negative pressure adhesion, biomimetic adhesion, and magnetic adhesion. Negative pressure adhesion climbing robots can adapt to various wall materials [7]. Biomimetic adhesion climbing robots achieve wall adhesion through mechanical devices such as claws, needles, and spikes [8] or through the use of adhesive materials between the robot and the wall [9]. Magnetic adhesion can be divided into two main types: electromagnetic adhesion and permanent magnet adhesion. The Spanish Institute of Industrial Automation developed an electromagnetic adhesion hexapod climbing robot with effective load of 100 kg. However, its own weight is as high as 220 kg, which prevents it from entering narrow and restricted spaces, making it impractical for on-site operations [10]. As for skid-steering technology, it is one of the most commonly used locomotion control methods [11]. The exploitation of the skid-steering method finds extensive applications across various domains, including the skid-steering control of climbing manipulation-oriented robots (CMo-Rs). A critical aspect of CMo-Rs involves trajectory tracking, as the tracking accuracy significantly impacts the overall reliability [12,13]. However, skid-steering control of CMo-Rs is challenging due to systematical nonlinear dynamics and the complex slip phenomenon.

Obviously, the essence of skid-steering control for a CMo-R is to improve its trajectory tracking ability. To tackle this problem, various trajectory-tracking techniques have been explored and can be broadly classified into geometric-based and model-based controllers. Geometric-based controllers guide the robot along a desired path by considering geometric relationships between its position, orientation, and reference trajectory. Examples include PID [14], pure pursuit [15], Stanley controller [16], and follow-the-carrot [17]. Model-based controllers rely on accurate mathematical models of robots to predict the response corresponding to control inputs and disturbances and generate appropriate control actions. Model-based controllers can be further categorized into two categories: (a) robust controllers, e.g., sliding mode controller (SMC) [18], adaptive controller [19], backstepping controller [20], H-infinity controller [21], and fuzzy logic [22], which ensure stability and satisfactory performance despite uncertainties. (b) Optimal controllers, e.g., model predictive controller (MPC) [23], linear quadratic regulator (LQR) [24], iterative learning controller [25], differential flatness-based controller [26], and nonlinear MPC [27] controller, which optimize specific performance criteria while adhering to constraints and system dynamics. Understanding the strengths and trade-offs of these trajectory-tracking techniques is essential for choosing the most suitable approach in climbing manipulation-oriented applications.

However, most of these methods are only suitable for mobile robots that move on the ground, while only a few of them involve control ability for climbing robotics, especially for climbing manipulation-oriented trajectory tracking. The ability to overcome slippage is the core feature that is different from common mobile robots. To enhance the ability of anti-slippage, various approaches, e.g., mechanical-based friction enhancement [4], tire-mass-based suction improvement [28], flexible adhesion strengthening [29], and terrain-adaptability-based magnetic optimization [30], etc., are proposed. Since climbing manipulation-oriented robotics are frequently used in industry operations, precision and security are of paramount importance. Although numerous classic control algorithms [31], advanced control methods [32], and artificial intelligence-based control frameworks [33] have been proposed recently, the comprehensive control method, which provides simple and robust yet effective anti-slippage ability, still needs further explorations.

The study focuses on airborne climbing and operation scenarios such as large-scale steel structures of ships, bridges, and petrochemical storage tanks, etc., aiming to investigate the impact of side slip on trajectory tracking for CMo-Rs. To achieve this, we developed a kinematic model that considers slip ratio and designed a simple yet reliable adaptive controller. With backstepping at the kinematics level, we were able to significantly reduce lateral deviation and improve the system stability and convergence rates during trajectory tracking, including on uncertain inclined planes. Furthermore, we were able to demonstrate asymptotic stability using the Lyapunov theory, effectively proving the stability of our proposed control system. Our approach was successfully tested through both simulation and experiments, demonstrating its applicability, feasibility, and stability. Overall, our study made four significant contributions: (1) a magnetic-adhesion-based mobile platform is designed for climbing manipulation with full-scenery climbing ability and redundant load-bearing mobility. (2) A simplified decoupling strategy, which transforms the originally complex climbing steering problem in 3D space into a planar skid-steering problem that only considers slip, is proposed for the skid-steering control. (3) An adaptive kinematic controller is developed for climbing manipulation on uncertain inclined planes based on skid-steering kinematics considering slip. (4) The control law for slip estimation is also designed, ensuring stable and robust tracking performance of CMo-Rs.

This paper is organized as follows: Section 2 outlines the mechanical structure of the CMo-R. Section 3 and Section 4 cover the skid-steering kinematics and controller design considering the slip. In Section 5, we implement the proposed kinematic controller and simulate it on different inclined planes with unknown tilt angles. Lastly, Section 6 presents the conclusions drawn from this study.

## 2. Mechanical Structure

### 2.1. Mechanical Design

**Figure 1:**
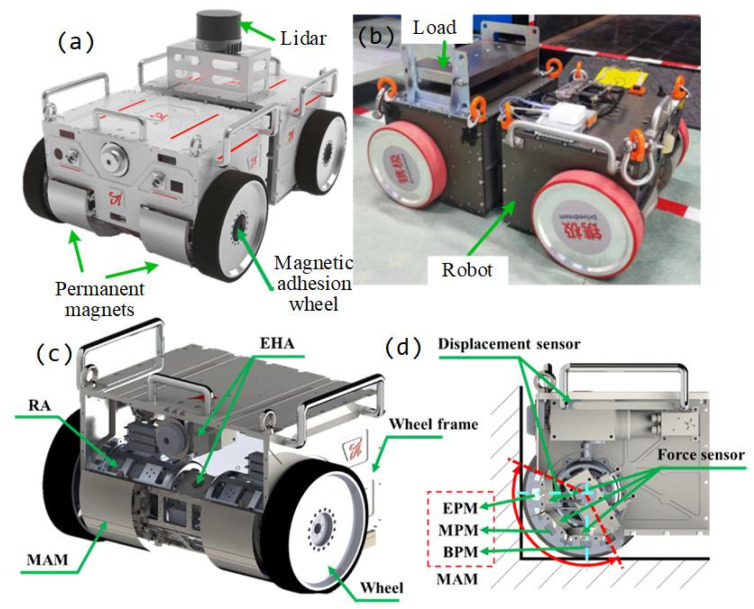
Mechanical structure of CMo-R. (**a**,**b**) Prototype of CMo-R; (**c**) Magnetic adhesion wheel and concealing front panel; (**d**) Magnetic adhesion wheel, showing one side wheel and rotary actuator.

The CMo-R under consideration is manufactured by Drivedream Machinery Equipment Co., Ltd in Shanghai. To ensure the CMo-R can move smoothly and complete the designated tasks on ferromagnetic surfaces, the mechanical structure of the robot mainly consists of two magnetic adhesion wheels, as shown in Figure 1a,b. The robot can carry 70 kg in weight and it is designed for climbing manipulation. In practice, mobility and trajectory-tracking performance are essential for climbing manipulation. For most cases, linear trajectory tracking is much more practical for climbing manipulation. Thus, linear trajectory tracking is paid more attention in this paper.

### 2.2. Magnetic Adhesion

**Figure 2:**
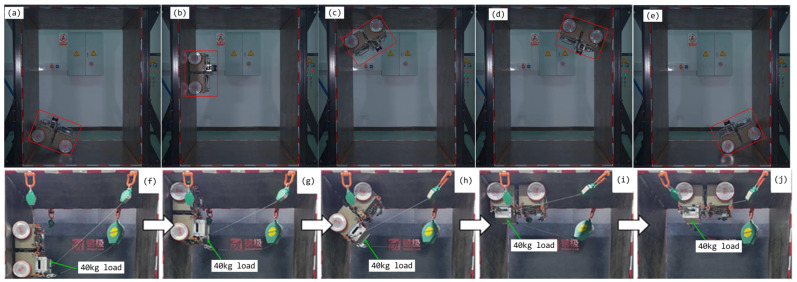
Full-scenery climbing ability and redundant load-bearing mobility. (**a**–**e**) full-scenery climbing without load; (**f**–**j**) full-scenery climbing with a 40 kg load.

The adhesion mechanism system utilizes permanent magnets to achieve full-scenery climbing ability and redundant load-bearing mobility. The magnetic adhesion wheel comprises various components, such as a wheel frame, wheels, rotary actuators (RAs), electro-hydraulic actuator (EHA), magnetic adhesion module (MAM), force sensor, and displacement sensor. For a better understanding of these components, please refer to Figure 2. The outer layer of the magnetic adhesion wheel is coated with polyurethane, while the inner layer is made of an aluminum alloy hub. The polyurethane coating directly contacts the wall surface to provide the necessary friction. The wheels are powered by 48 VDC and driven by RAs. The MAM comprises three groups of permanent magnets: end permanent magnets (EPMs), middle permanent magnets (MPMs), and bottom permanent magnets (BPMs). All magnetic groups are designed using the Halbach array and are located on the inner side of the wheels. The centers of the three groups of permanent magnets lie on the axis of the wheel, creating an enveloping angle of 135°, which ensures sufficient magnetic adhesion force on both sides of the internal angle of the walls. To ensure the safe movement of the robot, the magnetic force of each permanent magnet is constantly monitored in real time using force sensors.

### 2.3. Full-Scenery Climbing

The CMo-R boasts an innovative magnetic adhesion structure that allows it to climb any surface, even under a weight of up to 70 kg. As seen in Figure 2, the robot can move seamlessly from a horizontal to a vertical surface, crawl across a ceiling, and return to the ground. Its magnetic adhesion module design is ideal for traversing noncontinuous surfaces, making it a valuable tool for welding, polishing, rust removal, and other onboard climbing manipulations. However, its rigid structure limits its ability to adjust orientation on continuous surfaces, requiring precise skid-steering control. Subsequent sections will delve into the skid-steering control issue of the CMo-R.

### 2.4. Skid-Steering Control Architecture

**Figure 3:**
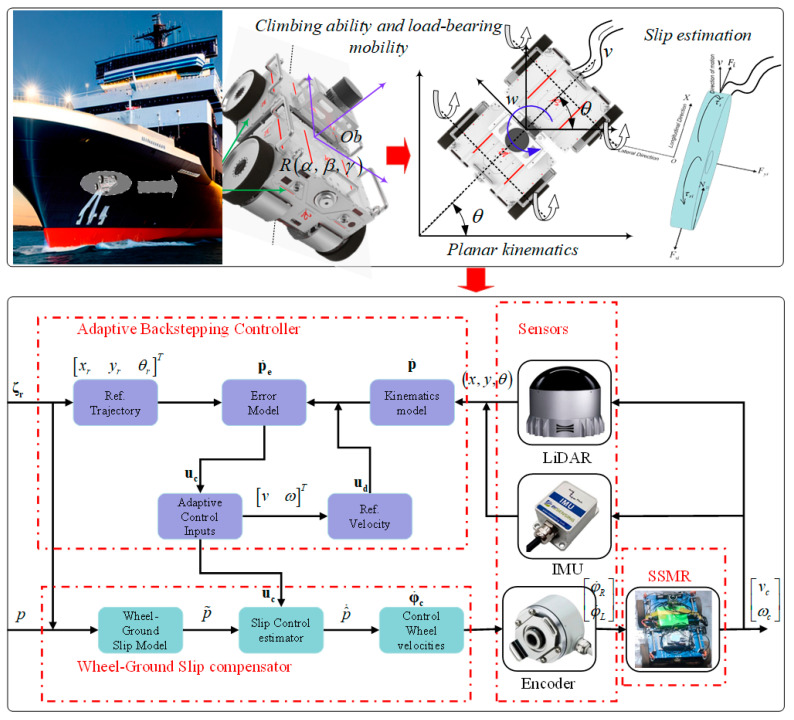
The simplified decoupling strategy for onboard climbing manipulation.

In Section 2.3, it was shown that the CMo-R has excellent wall-climbing capabilities and can bear heavy loads. Due to the low-speed and high-precision requirements of wall-climbing manipulations, the motion control of the CMo-R on three-dimensional surfaces is similar to that on the HP. However, there is a concern about slipping on the plane. To address this issue, a simplified decoupling strategy has been proposed in this paper for the skid-steering control system, as depicted in Figure 3. The strategy involves transforming the complex problem of wall-climbing steering in 3D space into a planar skid-steering problem that only considers the effects of slipping. This simplification allows for accurate control of wall-climbing steering using conventional algebraic methods and planar differential motion control models, without relying on complex theoretical tools, e.g., Lie groups/Lie algebra. It simplifies the overall control system by separating the problem of 3D wall-climbing control into the modeling of planar skid steering and control of planar trajectory tracking. Specifically, the modeling of planar skid steering takes slip effects into account. The control of planar trajectory tracking is explored in detail in the following sections.

## 3. Skid-Steering Kinematics

The CMo-R robot comprises three fundamental modules: the drive module, the control module, and the sensor module. These modules are interlinked and securely attached to the rigid body of the robot. The motion and stability of the robot are facilitated by four identical wheels, each driven by an individual DC-geared motor. It is postulated that all motors generate the same rotational speed at a given input voltage. In this section, we establish both the kinematic constraints and the slip-based kinematic model. Slippage can significantly impact the performance of a four-wheel skid-steering wall-climbing robot, especially when equipped with an airborne manipulator. Slippage occurs when the wheels lose traction with the surface on which the robot is moving, and this can have several effects:

- Reduced climbing efficiency: Slippage can decrease the robot’s ability to climb walls efficiently. If the wheels slip on the climbing surface, the robot may struggle to maintain a stable grip, making it difficult to ascend or descend the climbing wall smoothly.
- Loss of maneuverability: The ability to control and maneuver the robot is compromised when slippage occurs, especially when the airborne climbing manipulator is mounted. The external load affects the system’s efficacy, and the intended movements and commands cannot be accurately translated into actions if the wheels are slipping, leading to decreased overall performance.
- Decreased stability: Slippage can affect the stability of the robot. The airborne manipulator adds an additional layer of complexity, and any instability in the base can further complicate the control of the manipulator, hence design of an accurate control law is crucial. This is important for precise positioning and accurately tracking the trajectory.

This study addresses the incorporation of slippage effects into the robot kinematic model and emphasizes slip estimation in designing a robust controller. Furthermore, the design of the airborne manipulator considers the overall stability and control of the robot’s optimal performance.

### 3.1. Nonholonomic Kinematics Constraints

**Figure 4:**
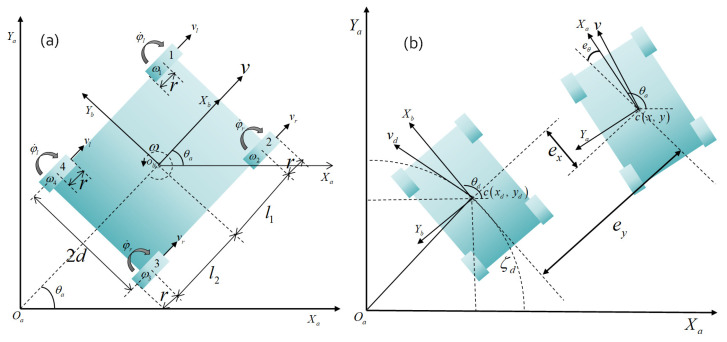
(**a**) Schematic representation of CMo-R; (**b**) Coordinate frame and error representation.

As illustrated in Figure 4a, a fixed global coordinate frame is established, denoted as $X_{a}O_{a}Y_{a}$, while the local coordinate frame fixed to the mobile robot is defined as $X_{b}O_{b}Y_{b}$, with its origin located at the robot’s center of mass. Key parameters include the separation between the wheels, denoted by 2*d*, and the wheelbase marked as *l* = *l*_1_ + *l*_2_. The radius of each wheel is represented by *r*. $\theta_{a}$ is the heading of the robot in the local coordinate frame with respect to the global coordinate frame. The linear and angular velocities of left and right side wheel pairs are respectively represented by $\left(v_{l},v_{r}\right)$ and $\left({\dot{\varphi }}_{l},{\dot{\varphi }}_{r}\right)$. The overall translational velocity of the nonholonomic robot system is *v* and the rotational velocity is ω.

The interrelation between the velocities of the wheels and those of the robot is defined in the following equations:

(1) $$\begin{array}{c} \left\{\begin{array}{c} {\dot{\varphi }}_{l}=\omega_{1}=\omega_{4}=\omega_{l}\\ {\dot{\varphi }}_{r}=\omega_{2}=\omega_{3}=\omega_{r} \end{array}\right.\\ v=\left(\frac{\omega_{r}+\omega_{l}}{2}\right)r\\ \omega =\left(\frac{\omega_{r}-\omega_{l}}{2d}\right)r \end{array}$$

Unlike conventional vehicles, the CMo-R achieves turning through differential speeds of its side wheel pairs. However, this differential speed control and the reduced traction in wheel–ground interactions can lead to a phenomenon known as ‘slip’ causing the wheels to slide instead of rolling. Consequently, controlling the motion of the CMo-R becomes challenging, particularly when navigating nonlinear paths. Therefore, we address this challenge by incorporating the slip factor into the kinematics model to enhance maneuverability. By considering the slip ratio of the right and left side wheel pairs, we aim to improve the robot’s ability to follow desired paths and achieve more precise motion control. The slip ratio $s_{i}$ is a crucial parameter that reflects the difference between the actual linear velocity of a wheel $r{\dot{\varphi }}_{i}$ and the effective linear velocity $v_{i}$ after slip. The slip ratio for the right and left side wheel pairs is defined as [34]:

(2) $$\left\{\begin{array}{c} s_{r}=\frac{r{\dot{\varphi }}_{r}-v_{r}}{r{\dot{\varphi }}_{r}}\\ s_{l}=\frac{r{\dot{\varphi }}_{l}-v_{l}}{r{\dot{\varphi }}_{l}} \end{array}∵0\leq s_{i}\leq 1\right.$$

where r is the wheel radius, ${\dot{\varphi }}_{r}$ and ${\dot{\varphi }}_{l}$ are the angular velocities of the right and left wheels. $v_{l}$ and $v_{r}$ are the corresponding linear velocities after slip. The slip parameter is expressed as

(3) $$\left\{\begin{array}{c} p_{r}=\frac{1}{1-s_{r}}\\ p_{l}=\frac{1}{1-s_{l}} \end{array}∵0\leq s_{i}\leq 1\right.$$

where $p_{l}$ and $p_{r}$ represent the left and right wheel pairs’ slip parameters. It is important to note that, in Equation (3), if the slip ratio $s_{i}$ becomes 1, the slip parameter $p_{i}$ will approach infinity. This implies that the wheel–ground traction is completely lost, and the control system may face challenges in accurately predicting and controlling the wheel’s motion producing effective linear velocity after slip $v_{i}$ is equal to zero, resulting in a scenario where the wheels are not moving relative to the ground. This situation indicates that the wheels are experiencing maximum skidding or slipping and no forward motion is produced by them. Therefore, it is crucial to maintain enough wheel–ground traction to minimize the slip effect.

This study employs a path-following controller to regulate the motion of the mobile robot, utilizing calculated translational and rotational velocities. A detailed discussion regarding the computation and implementation is presented in a subsequent section.

### 3.2. Kinematic Model Considering Slip

The positive quadrant of the geodetic coordinate system $\left\{X_{a},Y_{a},Z_{a}\right\}$, and the vehicle coordinate system $\left\{X_{b},Y_{b},Z_{b}\right\}$ are defined as shown in Figure 4b. The posture of the robot in the global frame $q_{a}\left(t\right)=\left[x_{a}\left(t\right),y_{a}\left(t\right),z_{a}\left(t\right)\right]$ represents the abscissa, ordinate, and heading with the control inputs $u\left(t\right)={\left[v\left(t\right),w\left(t\right)\right]}^{T}$ representing the linear and angular velocities. Let $q_{d}\left(t\right)=\left[x_{d}\left(t\right),y_{d}\left(t\right),z_{d}\left(t\right)\right]$ be the desired pose to follow the reference trajectory $\xi_{d}$ centered in the global coordinate frame, while the vector $e\left(t\right)=q_{a}\left(t\right)-q_{d}\left(t\right)$ describes the longitudinal, lateral, and yaw error, respectively. The reference [35] introduces the inverse kinematics model relationship for a 4-wheel skid-steering mobile robot. Notably, the existing model neglects the impact of wheel slippage, potentially influencing the controller’s overall performance. To address this limitation, the current study extends the inverse kinematics relationship, considering the effects of slip.

The kinematics model of the CMo-R with the slip parameter is formulated in the following equation:

(4) $$\dot{X}=R\dot{\varphi }=\left[\begin{array}{c} {\dot{X}}_{a}\\ {\dot{Y}}_{a}\\ {\dot{\theta }}_{a} \end{array}\right]=\left[\begin{array}{cc} \frac{r}{2p_{r}}\cos\theta_{a} & \frac{r}{2p_{l}}\cos\theta_{a}\\ \frac{r}{2p_{l}}\sin\theta_{a} & \frac{r}{2p_{l}}\sin\theta_{a}\\ \frac{r}{2dp_{r}} & -\frac{r}{2dp_{l}} \end{array}\right]\left[\begin{array}{c} {\dot{\varphi }}_{r}\\ {\dot{\varphi }}_{l} \end{array}\right]$$

If the velocity vector of the robot in the local coordinate is defined as $u\left(t\right)={\left[v\left(t\right),w\left(t\right)\right]}^{T}$, then the relation between φ and u including the slip parameter is derived as

(5) $$u=T\dot{\varphi }\Rightarrow \dot{\varphi }=T^{-1}u$$

where $u={\left[\begin{array}{ll} v(t) & \omega (t) \end{array}\right]}^{T};T=\left[\begin{array}{cc} \frac{r}{2p} & \frac{r}{2p}\\ \frac{r}{2dp} & -\frac{r}{2dp} \end{array}\right];\dot{\varphi }=\left[\begin{array}{ll} {\dot{\varphi }}_{Rd} & {\dot{\varphi }}_{Ld} \end{array}\right];T^{-1}=\frac{1}{r}\left[\begin{array}{cc} p_{r} & dp_{r}\\ p_{l} & -dp_{l} \end{array}\right]$. Finally, the relation of the right and left wheel pairs’ desired angular velocity with the influence of the slip parameter is given as

$$\left[\begin{array}{c} {\dot{\varphi }}_{Rd}\\ {\dot{\varphi }}_{Ld} \end{array}\right]=\frac{1}{r}\left[\begin{array}{cc} p & dp\\ p & -dp \end{array}\right]\left[\begin{array}{c} v\\ \omega \end{array}\right]$$

After inserting Equation (5) into (4), the simplified relation becomes

(6) $$\left[\begin{array}{c} {\dot{X}}_{a}\\ {\dot{Y}}_{a}\\ {\dot{\theta }}_{a} \end{array}\right]=\left[\begin{array}{cc} \cos\theta_{a} & 0\\ \sin\theta_{a} & 0\\ 0 & 1 \end{array}\right]\left[\begin{array}{c} v\\ \omega \end{array}\right]$$

Let $\dot{x}$, $\dot{y}$, and ω be the longitudinal, lateral, and angular velocities in the local coordinate frame, while the relation in the universal coordinate frame in the kinematics model is represented as

(7) $$\left[\begin{array}{c} {\dot{X}}_{a}\\ {\dot{Y}}_{a}\\ {\dot{\theta }}_{a} \end{array}\right]=\left[\begin{array}{c} \dot{x}\cos\theta_{a}-\dot{y}\sin\theta_{a}\\ \dot{x}\sin\theta_{a}+\dot{y}\cos\theta_{a}\\ \omega \end{array}\right]$$

For proper vehicle movement and control, it is imperative that the *x* component of the instantaneous center of rotation remains within the confines of the wheelbase, denoted as l1. If this condition is not met, the vehicle could potentially experience skidding in the y direction, leading to a loss of control. To ensure the vehicle’s smooth operation, the following relationship is established:

$$\left|-\frac{\dot{y}}{\omega }\right|<l_{1}\;\mathrm{or}\;\dot{y}=-b\omega ∵0<b<l_{1}$$

Inserting the effect of ICR in Equation (6), the overall kinematics model of the CMo-R skid-steering nonholonomic system becomes ${\dot{X}}_{a}=S(q)v$,

(8) $$\left[\begin{array}{c} {\dot{X}}_{a}\\ {\dot{Y}}_{a}\\ {\dot{\theta }}_{a} \end{array}\right]=\left[\begin{array}{cc} \cos\theta_{a} & \mathrm{bsin}\theta_{a}\\ \sin\theta_{a} & -\mathrm{bcos}\theta_{a}\\ 0 & 1 \end{array}\right]\left[\begin{array}{c} v\\ \omega \end{array}\right]$$

The above equation presents the kinematics model of a CMo-R, accounting for the effect of ICR and will be used in formulating the adaptive kinematics controller in the subsequent section. In this model, the control inputs are characterized by the wheel angular speeds and the slip ratio of the left and right wheel pairs. Then, we can obtain the following equation which encapsulates the representation of linear and angular velocity attributed to the rotation of each wheel, accounting for the slip parameter:

(9) $$\left\{\begin{array}{c} v=\frac{r}{2}\left(\frac{{\dot{\varphi }}_{r}}{p_{r}}+\frac{{\dot{\varphi }}_{L}}{p_{l}}\right)\\ \omega =\frac{r}{2d}\left(\frac{{\dot{\varphi }}_{r}}{p_{r}}-\frac{{\dot{\varphi }}_{L}}{p_{l}}\right) \end{array},∵\left(p_{r},p_{l}\right)\geq 1\right.$$

## 4. Controller Design for Trajectory Tracking

To control the CMo-R for better trajectory-tracking performance, the PID controller is one of the classic approaches. PID is a model-free method. The trajectory-tracking error is provided for feedback. However, the slip rate varies across different wheel–ground interactions due to differences in friction coefficients. Thus, both the PID controller and the adaptive kinematics controller are designed for comparison.

### 4.1. PID Controller

**Figure 5:**
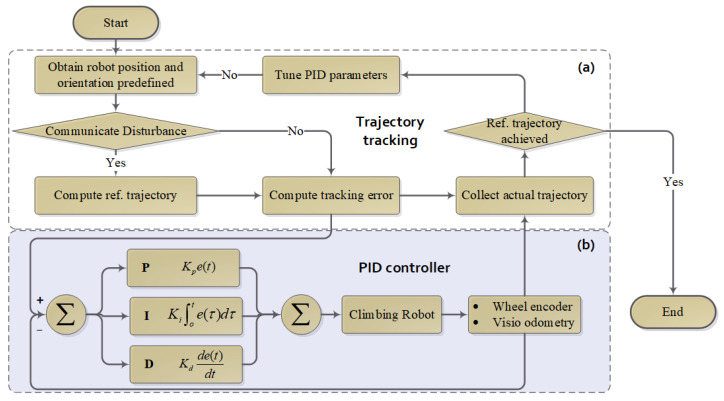
The architecture of PID-based trajectory-tracking controller; (**a**) Trajectory-tracking architecture; (**b**) PID controller.

The proportional–integral–derivative (PID) controller stands as the most prevalent type of closed-loop control system. These controllers operate by constantly measuring and modifying the system’s output to align with a predetermined set point, representing the desired condition for the system or process in question. What sets PID controllers apart is their adaptability, cost-effectiveness, and ease of implementation. They do not require extensive prior knowledge or a detailed system model, making them applicable in diverse fields ranging from hydraulics and pneumatics to both analog and digital electronics. In this paper, we designed a kinematics-level PID control algorithm to achieve smooth control. Proportional, integral, and derivative gains are introduced to tune the controller performance. The algorithm flowchart is shown in Figure 5.

Since the observation states are linear and angular velocities, $u={\left[\begin{array}{ll} v(t) & \omega (t) \end{array}\right]}^{T}$, then, the PID controller can be established as follows:

(10) $$\left\{\begin{array}{c} v\left(t\right)=K_{p}^{l}e_{l}\left(t\right)+K_{I}^{l}\int_{0}^{t}e\left(\tau \right)d\tau +K_{D}^{l}\frac{d}{dt}e\left(t\right)\\ \omega \left(t\right)=K_{p}^{a}e_{l}\left(t\right)+K_{I}^{a}\int_{0}^{t}e\left(\tau \right)d\tau +K_{D}^{a}\frac{d}{dt}e\left(t\right) \end{array}\right.$$

where $\left\{K_{p}^{l},K_{I}^{l},K_{D}^{l}\right\}$, $\left\{K_{p}^{a},K_{I}^{a},K_{D}^{a}\right\}$ are gain parameters for PID controllers of linear and angular velocity, respectively. Before the controllers are applied for trajectory tracking, the reference should be predefined according to different trajectories. In this paper, a circular trajectory is utilized for comparison.

### 4.2. Kinematic Controller

The first procedure is to determine a desired velocity control law that drives the tracking error between the current posture vector and the reference posture vector. A kinematic tracking error vector $e_{c}\left(t\right)$ and its time derivative ${\dot{e}}_{c}\left(t\right)$ are defined as

(11) $$p_{e}=\left[\begin{array}{c} X_{e}\\ Y_{e}\\ \theta_{e} \end{array}\right]=\left[\begin{array}{ccc} cos\theta_{a} & sin\theta_{a} & 0\\ -sin\theta_{a} & cos\theta_{a} & 0\\ 0 & 0 & 1 \end{array}\right]\left[\begin{array}{c} X_{d}-X_{a}\\ Y_{d}-Y_{a}\\ \theta_{d}-\theta_{a} \end{array}\right]$$

The relation for computing the time derivative of reference longitudinal, lateral, and yaw states in the global coordinate frame is given as ${\dot{X}}_{r}=S(q)v_{r}$.

(12) $$\left[\begin{array}{c} {\dot{X}}_{d}\\ {\dot{Y}}_{d}\\ {\dot{\theta }}_{d} \end{array}\right]=\left[\begin{array}{cc} \cos\theta_{d} & \mathrm{bsin}\theta_{d}\\ \sin\theta_{d} & -\mathrm{bcos}\theta_{d}\\ 0 & 1 \end{array}\right]\left[\begin{array}{c} v_{d}\\ \omega_{d} \end{array}\right]$$

We can find the time derivative of the above equation to find the ${\dot{e}}_{c}\left(t\right)$ matrix form in order to observe the variables’ relationship.

(13) $$\left[\begin{array}{c} {\dot{X}}_{e}\\ {\dot{Y}}_{e}\\ {\dot{\theta }}_{e} \end{array}\right]=\left[\begin{array}{c} {\dot{X}}_{d}\cos\left(\theta \right)-\dot{\theta }X_{d}\sin\left(\theta \right)-\dot{X}\cos\left(\theta \right)+\dot{\theta }X\sin\left(\theta \right)+{\dot{Y}}_{d}\sin\left(\theta \right)+\dot{\theta }Y_{d}\cos\left(\theta \right)-\dot{Y}\sin\left(\theta \right)-\dot{\theta }Y\cos\left(\theta \right)\\ -{\dot{X}}_{d}\sin\left(\theta \right)-\dot{\theta }X_{d}\cos\left(\theta \right)+\dot{X}\sin\left(\theta \right)+\dot{\theta }X\cos\left(\theta \right)+{\dot{Y}}_{d}\cos\left(\theta \right)-\dot{\theta }Y_{d}\sin\left(\theta \right)-\dot{Y}\cos\left(\theta \right)+\dot{\theta }Y\sin\left(\theta \right)\\ {\dot{\theta }}_{d}-\dot{\theta } \end{array}\right]$$

Substituting Equations (8) and (12) in (13) and simplifying the relation yields

(14) $$\left[\begin{array}{c} {\dot{X}}_{e}\\ {\dot{Y}}_{e}\\ {\dot{\theta }}_{e} \end{array}\right]=\left[\begin{array}{c} \omega Y_{e}-v+v_{d}cos\left(\theta_{e}\right)+b\omega_{d}sin\left(\theta_{e}\right)\\ v_{d}sin\left(\theta_{e}\right)-b\omega_{d}cos\left(\theta_{e}\right)+\omega \left(b-X_{e}\right)\\ \omega_{d}-\omega \end{array}\right]$$

The Lyapunov function is introduced using the above concept, where $k_{2}\geq 0$ is a constant parameter greater than zero. It is obvious that the function V≥0, if and only if ${\left[x_{e},y_{e},\theta_{e}\right]}^{T}=0$, then V=0. The Lyapunov function is selected as

(15) $$V_{1}=\frac{1}{2}X_{e}^{2}+\frac{1}{2}Y_{e}^{2}+\frac{\left(1-cos\left(\theta_{e}\right)\right)}{k_{2}}>0$$

The time derivative of the Lyapunov function should be negative definite which ensures that the control law is stable over the infinite period which means the system is asymptotically stable.

(16) $${\dot{V}}_{1}=X_{e}{\dot{X}}_{e}+Y_{e}{\dot{Y}}_{e}+{\dot{\theta }}_{e}\frac{sin\left(\theta_{e}\right)}{k_{2}}<0$$

After inserting the values of ${\dot{X}}_{e},{\dot{Y}}_{e},{\dot{\theta }}_{e}$, from Equation (14), we obtain

(17) $${\dot{V}}_{1}=X_{e}\left(\omega Y_{e}-v+v_{d}cos\left(\theta_{e}\right)+b\omega_{d}sin\left(\theta_{e}\right)\right)+Y_{e}\left(v_{d}\sin\left(\theta_{e}\right)-b\omega_{d}\cos\left(\theta_{e}\right)+\omega \left(b-x_{e}\right)\right)$$

After simplifying the above equation, we formulate the following control law for linear and angular velocity:

(18) $$\left[\begin{array}{c} v\\ \omega \end{array}\right]=\left[\begin{array}{c} v_{d}cos\left(\theta_{e}\right)+b\omega_{d}sin\left(\theta_{e}\right)+k_{1}X_{e}\\ \frac{1}{\left(1-k_{2}Y_{e}bcsc\left(\theta_{e}\right)\right)}\left(\omega_{d}+k_{2}Y_{e}v_{d}-k_{2}Y_{e}b\omega_{d}cot\left(\theta_{e}\right)+k_{3}sin\left(\theta_{e}\right)\right) \end{array}\right]$$

The above equation represents the control law for the skid-steering mobile robot with $k_{1},\;k_{2}$, and $k_{3}$ as the gain parameters to tune the controller. Substituting Equation (18) to (17), one can obtain

(19) $${\dot{V}}_{1}={{-k_{1}X}_{e}}^{2}-\frac{k_{3}\sin\theta_{e}}{k_{2}}\leq 0$$

The conditions $V_{1}\geq 0$ and ${\dot{V}}_{1}\leq 0$ imply that as t→∞, $p_{e}$ tends to zero. This behavior indicates that the control law given by Equation (18) is asymptotically stable.

### 4.3. Adaptive Slip Control

The validity of the control law given in Equation (18) implies the absence of slip during wheel–ground interactions. Unlike conventional vehicles, where steering angles are responsible for the wheel turn, skid-steering mobile robots achieve rotation by rotating their outer wheels at a higher speed than the inner wheels. However, the occurrence of slip can significantly impact overall turning accuracy. Thus, it is imperative to account for slips in the design and implementation of these control systems. If the parameter s in Equation (2) is unknown, the slip parameter in Equation (3) cannot be determined. Consequently, Equation (7) is invalid.

To facilitate the computation of the updated law, slip parameter estimation becomes essential. We assume that the slip on each side of the wheel pair is identical, denoted as $s_{r}=s_{l}=s$ and $p=\frac{1}{1-s}$. The error derivatives with respect to time can be written in state-space form:

(20) $$\left[\begin{array}{c} {\dot{X}}_{e}\\ {\dot{Y}}_{e}\\ {\dot{\theta }}_{e} \end{array}\right]=\left[\begin{array}{cc} cos\theta_{e} & bsin\theta_{e}\\ sin\theta_{e} & -bcos\theta_{e}\\ 0 & 1 \end{array}\right]\left[\begin{array}{c} v_{d}\\ \omega_{d} \end{array}\right]+\left[\begin{array}{cc} -1 & y_{e}\\ 0 & b-x_{e}\\ 0 & -1 \end{array}\right]\left[\begin{array}{c} v\\ \omega \end{array}\right]$$

Putting Equation (5) in (20), we obtain

(21) $$\left[\begin{array}{c} {\dot{X}}_{e}\\ {\dot{Y}}_{e}\\ {\dot{\theta }}_{e} \end{array}\right]=\left[\begin{array}{cc} cos\theta_{e} & bsin\theta_{e}\\ sin\theta_{e} & -bcos\theta_{e}\\ 0 & 1 \end{array}\right]\left[\begin{array}{c} v_{d}\\ \omega_{d} \end{array}\right]+\left[\begin{array}{cc} -1 & Y_{e}\\ 0 & b-X_{e}\\ 0 & -1 \end{array}\right]\left[\begin{array}{cc} \frac{r}{2p} & \frac{r}{2p}\\ \frac{r}{2dp} & -\frac{r}{2dp} \end{array}\right]\left[\begin{array}{c} {\dot{\varphi }}_{Rd}\\ {\dot{\varphi }}_{Ld} \end{array}\right]$$

The constant slip parameter mentioned in the above equation is unknown, so the relation for Equation (5) is undetermined. To handle this problem, we need to introduce the estimation $\hat{p}$ of p. Replacing the slip parameter to estimate slip in Equation (7), we can write

(22) $${\dot{\varphi }}_{d}=\left[\begin{array}{c} {\dot{\varphi }}_{Rd}\\ {\dot{\varphi }}_{Ld} \end{array}\right]=\frac{1}{r}\left[\begin{array}{cc} \hat{p} & d\hat{p}\\ \hat{p} & -d\hat{p} \end{array}\right]\left[\begin{array}{c} v\\ \omega \end{array}\right]=\frac{1}{r}{\hat{T}}^{-1}u$$

where ${\dot{\varphi }}_{d}={\left[\begin{array}{cc} {\dot{\varphi }}_{Rd} & {\dot{\varphi }}_{Ld} \end{array}\right]}^{T}$ represents the desired angular velocity of the right and left side wheel pairs, respectively. Additionally, $u={\left[\begin{array}{cc} v & \omega \end{array}\right]}^{T}$ denotes the control linear and angular velocities after slip estimation, respectively. By substituting Equation (22) into Equation (21), we obtain

(23) $$\left[\begin{array}{c} {\dot{X}}_{e}\\ {\dot{Y}}_{e}\\ {\dot{\theta }}_{e} \end{array}\right]=\left[\begin{array}{cc} cos\theta_{e} & bsin\theta_{e}\\ sin\theta_{e} & -bcos\theta_{e}\\ 0 & 1 \end{array}\right]\left[\begin{array}{c} v_{d}\\ \omega_{d} \end{array}\right]+\left[\begin{array}{cc} -1 & Y_{e}\\ 0 & b-X_{e}\\ 0 & -1 \end{array}\right]\left[\begin{array}{cc} \frac{\hat{p}}{p} & 0\\ 0 & -\frac{\hat{p}}{p} \end{array}\right]\left[\begin{array}{c} v\\ \omega \end{array}\right]$$

Since the left side of Equation (23) represents the error rate in position and orientation of the robot, we have to define the error for slip estimation and that is $\tilde{p}=p-\hat{p}$. Replacing $\hat{p}$ in the above equation to represent in terms of $\tilde{p}$ is as follows:

(24) $$\left[\begin{array}{c} {\dot{X}}_{e}\\ {\dot{Y}}_{e}\\ {\dot{\theta }}_{e} \end{array}\right]=\left[\begin{array}{cc} cos\theta_{e} & bsin\theta_{e}\\ sin\theta_{e} & -bcos\theta_{e}\\ 0 & 1 \end{array}\right]\left[\begin{array}{c} v_{d}\\ \omega_{d} \end{array}\right]+\left[\begin{array}{cc} -1 & Y_{e}\\ 0 & b-X_{e}\\ 0 & -1 \end{array}\right]\left[\begin{array}{cc} 1-\frac{\tilde{p}}{p} & 0\\ 0 & 1-\frac{\tilde{p}}{p} \end{array}\right]\left[\begin{array}{c} v\\ \omega \end{array}\right]$$

To derive an updated law for computing the slip estimation $\hat{p}$, the following Lyapunov function is introduced:

(25) $$V_{2}=V_{1}+\frac{{\tilde{p}}^{2}}{2\beta p}>0$$

In which slip constant p≥1 and slip gain β>0. The derivative of the above Lyapunov function should be negative definite in order to ensure the control law stability and has the following relation:

(26) $${\dot{V}}_{2}=X_{e}{\dot{X}}_{e}+Y_{e}{\dot{Y}}_{e}+{\dot{\theta }}_{e}\frac{\sin\left(\theta_{e}\right)}{k_{2}}+\frac{\tilde{p}\dot{\tilde{p}}}{\beta p}<0$$

Inserting the error values from (21) and simplifying, one can obtain

(27) $${\dot{V}}_{2}=X_{e}\left[-v+v_{d}cos\left(\theta_{e}\right)+b\omega_{d}sin\left(\theta_{e}\right)\right]+\;\frac{sin(\theta_{e})}{k_{2}}\left[k_{2}Y_{e}v_{d}-k_{2}Y_{e}b\omega_{d}\cot\left(\theta_{e}\right)+k_{2}Y_{e}b\omega cos\left(\theta_{e}\right)+\omega_{d}-\omega \right]-\;\frac{\tilde{p}}{p}\left[\frac{\hat{p}}{\beta }-vX_{e}-\omega \left\{\frac{k_{2}X_{e}Y_{e}+k_{2}Y_{e}\left(b-x_{e}\right)-sin(\theta_{e})}{k_{2}}\right\}\right]$$

where $\dot{\tilde{p}}$ = $-\dot{\hat{p}}$.

To ensure the stability of the control law, ${\dot{V}}_{2}\leq 0$ guarantees the system is stable. Hence, the estimated control law after simplifying Equation (25) is

(28) $$\left[\begin{array}{c} v\\ \omega \end{array}\right]=\left[\begin{array}{c} v_{d}cos\left(\theta_{e}\right)+b\omega_{d}sin\left(\theta_{e}\right)+k_{1}X_{e}\\ \frac{\left(\omega_{d}+k_{2}Y_{e}v_{d}-k_{2}Y_{e}b\omega_{d}cot\left(\theta_{e}\right)+k_{3}sin\left(\theta_{e}\right)\right)}{\left(1-k_{2}y_{e}bcsc\left(\theta_{e}\right)\right)} \end{array}\right]$$

Hence, the kinematic adaptive control law vector for the system with an unknown slip parameter in Equation (26) is identical to the kinematic control law vector for the system without an unknown slip parameter in Equation (16). The update law for estimating $\dot{\hat{p}}$ can be formulated as follows:

(29) $$\dot{\hat{p}}=\beta \left[vx_{e}-\omega \left\{\frac{k_{2}x_{e}y_{e}+k_{2}y_{e}\left(b-x_{e}\right)-\sin\left(\theta_{e}\right)}{k_{2}}\right\}\right]$$

Equation (29) represents the control law designed for slip estimation, ensuring stable and robust tracking performance along a nonlinear trajectory for a nonholonomic system.

### 4.4. Stability Analysis

According to the adaptive backstepping method, the time derivative of the Lyapunov function for the CMo-R is

(30) $${\dot{V}}_{2}=-k_{1}X_{e}^{2}-\frac{k_{3}\sin\theta_{e}^{2}}{k_{2}}-\frac{\tilde{p}}{p}\left(0\right)\leq 0$$

Because ∀t∈[0,+∞), it is concluded that $v_{d}$ and $\omega_{d}$ are bounded, and ${\left[\begin{array}{ccc} X_{e} & Y_{e} & \theta_{e} \end{array}\right]}^{T}=0$ are uniformly bounded. This is because $k_{1},{\;k}_{2},\;\mathrm{and}\;k_{3}$ are constants greater than zero, and $k_{3}\sin\theta_{e}^{2}/k_{2}$ is positive definite for any value of $\theta_{e}$ in the domain as $D=\left\{e(t)\in R^{3}Ι\right.-\pi \leq \theta_{e}\leq \left.\pi \right\}$. It can be summed up that $V_{2}$ is a positive definite continuous and bounded differentiable function while ${\dot{V}}_{2}$ is a negative definite uniformly continuous function under the defined condition of ∀t∈[0,+∞) in a domain D. This implies that $V_{2}$ is a nonincreasing function that converges to some constant positive value and hence the error ${\left[\begin{array}{ccc} X_{e} & Y_{e} & \theta_{e} \end{array}\right]}^{T}$ and the estimation parameter $\tilde{p}$ are bounded. Given that $u_{d}={\left[\begin{array}{cc} v_{d} & \omega_{d} \end{array}\right]}^{T}$ is bounded and hence $u={\left[\begin{array}{cc} v & \omega \end{array}\right]}^{T}$ is also uniformly bounded, $\dot{e}(t)$ in Equation (24) is bounded.

According to the Barbalat lemma, when $t\to \infty ,V_{2}$, a noninceasing function, converges to some positive constant value making ${\dot{V}}_{2}\to 0$, implying ${\left[\begin{array}{ccc} X_{e} & Y_{e} & \theta_{e} \end{array}\right]}^{T}$ should converge to zero, respectively, at the equilibrium point. Referring to Equation (29), as $\left\{x_{e},\left.\theta_{e}\right\}\right.\to 0$ makes the $\hat{p}$ nonincreasing, thereby making the overall Equation (29) uniformly bounded as the following equation:

(31) $$\underset{t\to \infty }{lim}p_{e}=\underset{t\to \infty }{lim}\left[\left|X_{e}(t)\right|+\left|Y_{e}(t)\right|+\left|\theta_{e}\left(t\right)\right|\right]=0$$

This analysis demonstrates that the pose error of the closed-loop control system, as described in Equation (31), possesses characteristics that render the controller asymptotically stable within the defined domain *D* based on the calculated values.

### 4.5. Comparative Analysis of Adaptive Kinematic and PID Controllers

The adaptive kinematic control algorithm based on slip estimation and Lyapunov theory offers several advantages over the traditional PID controller in the context of controlling a CMo-R skid-steering nonholonomic system with wheel slip. The adaptive kinematic controller offers adaptability, robustness, stability guarantees, and improved performance in scenarios with wheel slip compared to the traditional PID control algorithm. These advantages make it particularly suitable for systems where slip is a critical factor, such as four-wheel vehicles navigating challenging terrains. The comparison between the controllers shown in Table 1.

## 5. Experiment and Simulation

In this section, both the PID controller and the adaptive kinematic controller are implemented. Specifically, the adaptive kinematic controller considers different slip factors, which are simulated and experimented with on uncertain inclined planes. The results of numerical simulations and on-site experiments provide evidence for the effectiveness of this control method, demonstrating precise control of slip turning for mobile robots on uncertain inclined planes.

### 5.1. PID-Based Control

For comparison, the PID controller is implemented and simulated with the following hardware configuration and parameter list.

**Figure 6:**
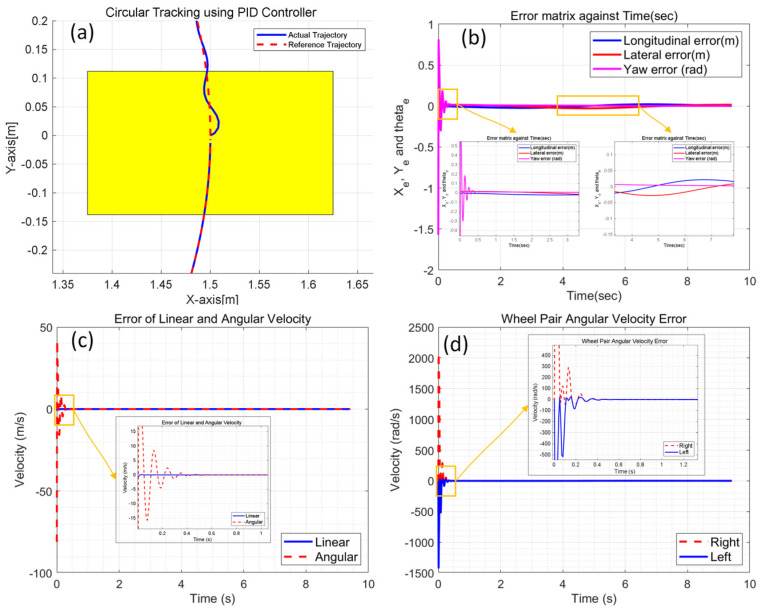
Simulation results of circular trajectory tracking using PID controller; (**a**) local details of circular trajectory; (**b**) longitudinal, lateral, and yaw error; (**c**) linear velocity and angular velocity tracking error; (**d**) angular velocity errors of right and left wheel pair.

For simplification, the CMo-R is represented as a four-wheel skid-steering robot considered for circular trajectory tracking. Parameters are set according to Table 2. In this simulation, the CMo-R is controlled to track a circular trajectory. As shown in Figure 6a, the local details of the initial tracking performance may be affected by proportional gain, integral gain, and derivative gain. And the tracking trajectory is oscillating in the first second. According to Figure 6b, the oscillation is mainly produced in the yaw dimension in the 1st second, and the oscillation is also shown in angular velocity errors, which are shown in Figure 6c. Then, the oscillation is restrained by tuning the position and orientation from 4 s to 6 s. Accurately, the controller is tuning all the time until both the linear and angular velocities reach the steady state. The velocities of the right wheel pair and left wheel pair are adjusted based on the proportional term, integral term, and derivative term. Considering the kinematic characteristic of anti-clockwise circle motion, the amplitude of the right pair is approximately 2000 rad/s, which is much larger than that of the left side (1500 rad/s). The velocities of both sides will reach an equilibrium state finally.

### 5.2. Adaptive Kinematic Control

The proposed method utilizes a backstepping algorithm for adaptive kinematic control. Except for the mechanical parameters, some assistant hyperparameters are also supplied in this kinematic control algorithm, and the details are listed in the following Table 3.

**Figure 7:**
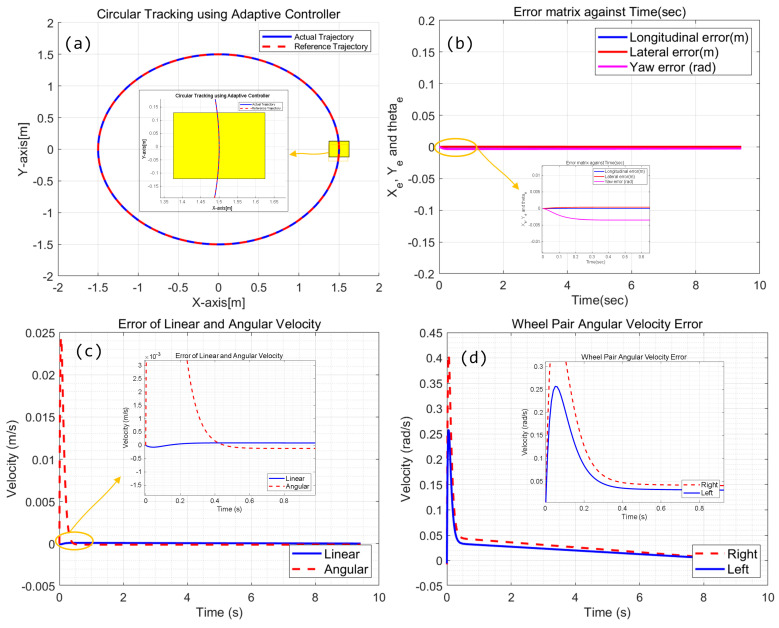
Simulation results of circular trajectory tracking using adaptive controller; (**a**) tracking trajectory with local information; (**b**) Error of position and orientation; (**c**) Error of linear and angular velocities; (**d**) angular velocity errors of right and left wheel pair.

Based on the above configuration, numerical simulation for adaptive kinematic control of the CMo-R is carried out. Compared with the PID controller, the performance of circular tracking is much smoother even in the initial state. There is no oscillation during the tracking period, as shown in Figure 7a,b. The controller produces better performance. Longitudinal and lateral errors are less than 0.5 mm. However, the yaw error is much larger. There exists a constant latency difference between the reference velocity and actual velocity, as shown in Figure 7c, which reduces the tracking performance of angular velocity.

On the other side, considering the slip factors, the velocity difference between the left wheel pair and right wheel pair, which is demonstrated in Figure 7d, also deteriorates the performance. At the initial stage, large acceleration is applied on the right pair of wheels to keep up with the change in angular velocity. Due to maximum accelerating ability, the desired velocity may cost more time to track. As a result, it produces a constant tracking error.

**Figure 8:**
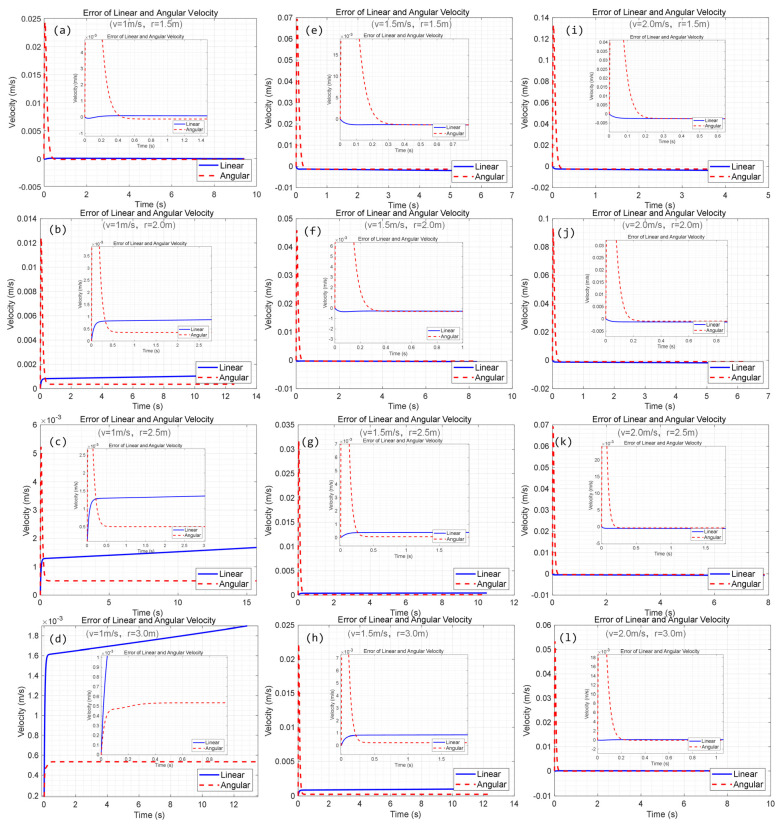
The influence of trajectory curvature and tracking velocity on tracking performance. (**a**–**d**) Simulation results with various tracking radiuses when *v* = 1.0 m/s; (**e**–**h**) simulation results with various tracking radiuses when *v* = 1.5 m/s; (**i**–**l**) simulation results with various tracking radiuses when *v* = 2.0 m/s.

During the simulation, both the tracking velocity and trajectory curvature affect the final tracking performance. Aiming at the influence of trajectory curvature and tracking velocity on performance, simulations are also carried out. Results are shown in Figure 8.

It can be seen from Figure 8 that when the robot moves at a speed of 1 m/s, the tracking error of the linear velocity and the angular velocity decreases as the curvature of the reference trajectory increases. This is because the greater the curvature of the trajectory, the greater the probability of slip during skid steering, which increases the tracking error. When the reference trajectory radius is R = 3 m, the RMSE of the angular velocity is approximately 0.47 × 10^−^³. The primary error comes from the initial stage of the motion, which keeps up with the change in the angular velocity at the fastest speed. While the change in the linear velocity is relatively stable, the RMSE is approximately 1.8 × 10^−^³. The trajectory tracking error is less than 2 mm.

**Figure 9:**
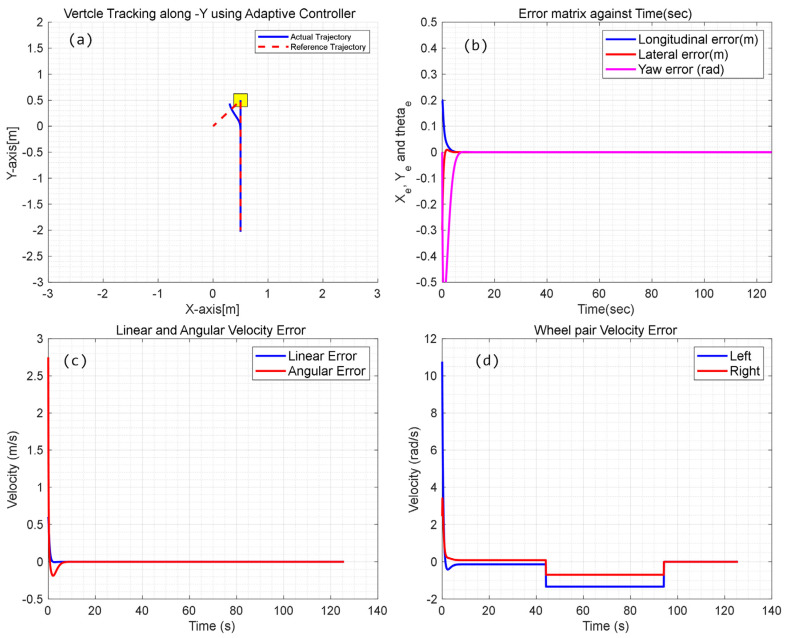
Simulation results of linear trajectory tracking with vertical orientation; (**a**) global view of tracking trajectory; (**b**) Error of position and orientation; (**c**) Error of linear and angular velocities; (**d**) angular velocity errors of right and left wheel pair.

On the other hand, if the radius of the reference trajectory remains constant, both the linear velocity and angular velocity tracking errors tend to increase as the velocity increases. According to the average value of RMSE, the tracking error is maintained below 5 mm, and the tracking error for both linear and angular velocity is 1.33 mm/s, even in the worst case, i.e., the linear velocity is 2 m/s and trajectory curvature is 1.5 m. The error is primarily attributed to the initial stage of motion adjustment, as shown in Figure 9a. Therefore, in practice, for better skid-steering control of the CMo-R, a smaller velocity and gentler path should be chosen for climbing manipulation, e.g., welding, grinding, and other scenes. Theoretically, the longer the path traveled, the smaller the average error generated. This is because the main error generated during the initial adjustment phase is reduced.

### 5.3. Climbing Manipulation-Oriented Linear Trajectory Tracking

The most significant difference between climbing tasks and regular planar motion tasks lies in the continuous control of the body posture. This control algorithm involves complex geometric motion modeling, e.g., Lie groups/Lie algebras, with significant theoretical depth and computational complexity. However, for climbing tasks such as welding and polishing, which involve movement on vertical surfaces, the control of the mobile platform requires higher tracking performance for a linear trajectory. Therefore, this section focuses on the simulation and experimentation of linear trajectory tracking required for climbing tasks such as welding and polishing.

**Figure 10:**
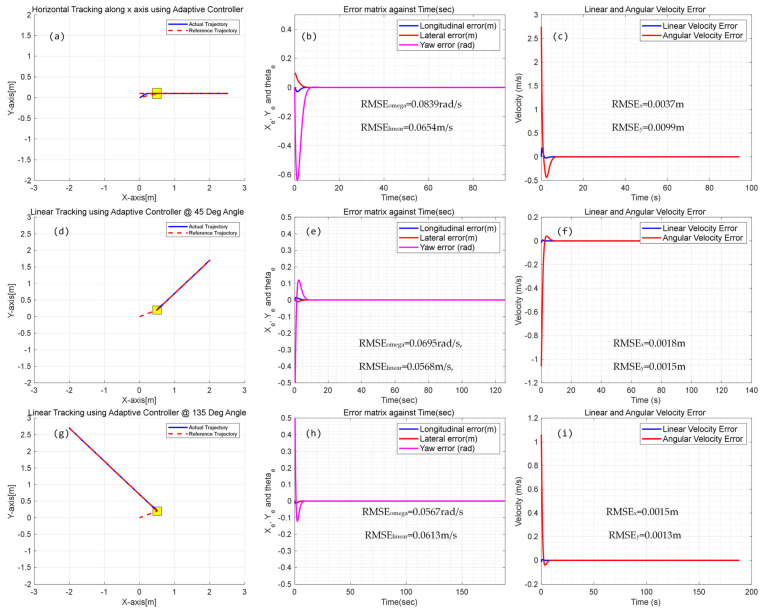
Simulation results of linear trajectory tracking with different orientations; (**a**–**c**) Tracking trajectory, position, and orientation error and velocity tracking error of simulation with horizontal orientation; (**d**–**f**) Tracking trajectory, position, and orientation error and velocity tracking error of simulation with 45° orientation; (**g**–**i**) Tracking trajectory, position, and orientation error and velocity tracking error of simulation with 135° orientation.

In linear trajectory tracking, we have implemented a motion control algorithm that considers only sliding without delving into differential geometry. We conducted simulation analysis in four different poses, namely, along the y-axis, x-axis, 45° diagonal, and 135° diagonal direction for repeated forward and backward linear trajectory tracking. The results are shown in Figure 9 and Figure 10. During the simulation, the CMo-R was controlled to move forward and backward. As shown in Figure 9, due to the initial position being different from the predetermined reference, the CMo-R deviates significantly from the intended trajectory. However, through algorithm adaptation, the CMo-R reaches the desired trajectory after an adjustment time of 2 to 5 s.

Additionally, various initial poses were analyzed through simulation in three different directions, along the y-axis, 45° diagonal, and 135° diagonal. Figure 10 illustrates the corresponding trajectory tracking errors, actual linear and angular velocity errors, and velocity tracking errors of the left and right drive wheels of the CMo-R. The angular velocity, linear velocity, and position errors along the x- and y-axes of the CMo-R were statistically analyzed through repeated simulations. Table 4 summarizes the mean square error (MSE) values obtained from the analyses.

### 5.4. Experiment Configuration

The study aimed to investigate the linear trajectory tracking of a climbing manipulation system on various inclined planes. A physical prototype with a maximum linear speed of 2 m/s and a minimum motion curvature radius of 0.5 m was utilized to test the proposed method, which was found to be fully applicable to the controller. The CMo-R was deployed in a custom-designed experimental cabin model with a height of 12 m, and its performance was evaluated on a horizontal plane (HP), 50° inclined plane (50° IP), 60° inclined plane (60° IP), and vertical plane (VP) with magnetic adsorption. The experimental results are presented in Table 4, along with images of the CMo-R prototype in each scenario. This research provides valuable insights into the capabilities and limitations of climbing manipulation systems for navigation on inclined planes, which can inform future development efforts aimed at improving their performance and safety.

Furthermore, to ensure accurate feedback and ground truth positioning of the CMo-R within the experimental cabin, a real 3D model of the cabin is utilized to generate a point cloud. The positioning of the CMo-R was achieved through the utilization of a Livox lidar sensor and the implementation of the adaptive Monte Carlo localization (AMCL) algorithm. The comprehensive configuration is shown in Table 5.

Considering the high requirements for linear trajectory-tracking performance on various inclined planes in climbing tasks such as welding and polishing, the following section demonstrates real-world experiments on an HP, 50° IP, 60° IP, and VP with magnetic adsorption.

### 5.5. Experiment Results

Considering the precision and anti-slip technology requirements for climbing operations, it is necessary to conduct more refined experimental research on linear tracking for wall-climbing operations. Therefore, experiments are conducted on an HP, 50° IP, 60° IP, and VP. These experiments demonstrate the effectiveness of the proposed approach in achieving high precision and stability in challenging environments.

**Figure 11:**
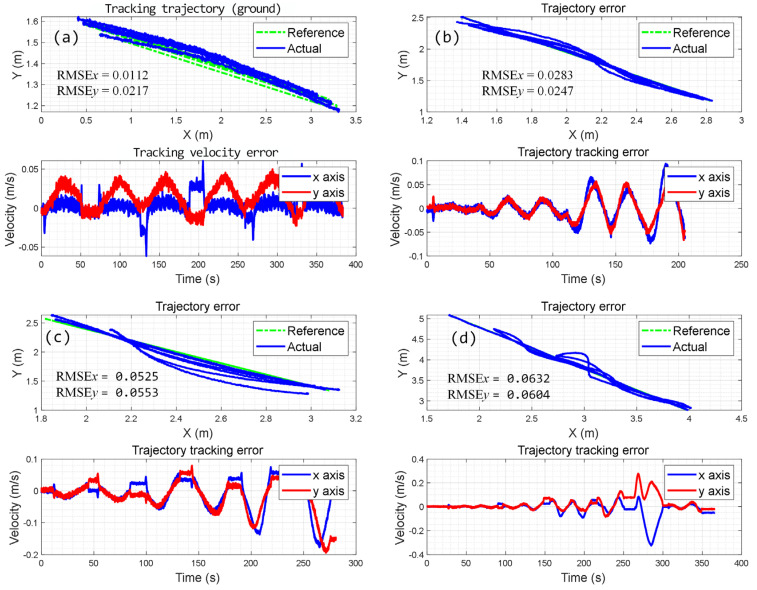
Experiment results on various inclined planes without slip estimation; (**a**) on the ground; (**b**) results on 50° IP; (**c**) results on 60° IP; (**d**) results on VP.

The experiment is performed by controlling the robot moving back and forth without considering the sliding effect. Results are shown in Figure 11a, where it can be observed that the tracking error in the x-axis direction reaches 11.2 mm, while the error in the y-axis direction exceeds 20 mm. This level of error is unacceptable in practical applications.

The proposed approach has also been validated for climbing on sloped terrains with magnetic adsorption. To validate its effectiveness on surfaces with different slip rates, experimental research was conducted on two differently inclined planes (IPs) with magnetic adsorption. When controlled by kinematics without considering slip, the experimental results on the 50° IP and 60° IP were significantly different, as shown in Figure 11b,c. For example, when performing linear tracking along different orientations on the 50° IP, it was found that the root mean square error (RMSE) is 28.3 mm in the x-axis direction and 24.7 mm in the y-axis direction. However, when experiments were conducted on the 60° IP, the results were 52.5 mm in the x-axis direction and 55.3 mm in the y-axis direction. Finally, the experiment is also performed on a VP, as shown in Figure 11d. The error was larger when the influence of slip estimation was not considered, with an RMSE of 60 mm. The influence of slipping renders this approach unusable in practical engineering scenarios.

**Figure 12:**
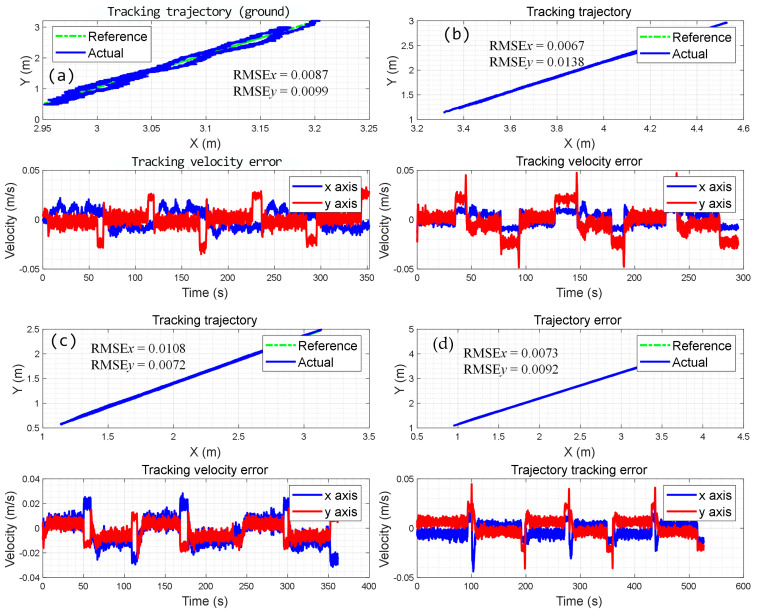
Experiment results on various inclined planes with slip estimation; (**a**) on the ground; (**b**) results on 50° IP; (**c**) results on 60° IP; (**d**) results on VP.

By utilizing the proposed adaptive kinematic controller that considers the slip rate, another set of linear trajectory-tracking experiments was conducted, and the results are shown in Figure 12. When performed on an HP, the tracking errors in the x-axis and y-axis directions are 8.7 mm and 9.9 mm, respectively. It has met the accuracy requirements of most operations, especially in the field of climbing manipulation. Then, the proposed adaptive kinematic control method is also verified on 50° IP and 60° IP, as shown in Figure 12b,c, and it exhibits a certain level of effectiveness in suppressing slip, with a minimum error of only 6 mm. Similar results are achieved in the experiments conducted on a VP, as shown in Figure 12d, and the minimum error is only 6.7 mm. Furthermore, the velocity navigation errors in the x-axis and y-axis directions are negligible, with error peaks at only 0.1% of the travel speed. This means that the adaptive skid-steering control approach can be applied to inclined planes without knowing the tilt angle. The proposed method, with its simple framework and relatively stable performance, demonstrates good applicability in the field of wall-climbing manipulation.

As shown in Table 6, the performance of linear trajectory tracking using the proposed method is represented by RMSE. The data in the table indicate that the algorithm that considers slippage produces significantly more accurate results than the algorithm that does not consider slippage. In fact, in the worst-case scenario, the algorithm that considers slippage reduces the error caused by slippage by 30%. Therefore, it can be concluded that the control effect after slip estimation is superior.

## 6. Conclusions

This study aimed to address the slipping phenomenon that occurs when a CMo-R turns. To achieve this, a four-wheel kinematic model that considers slip rate effects was constructed, and an adaptive kinematic control algorithm based on Lyapunov theory was designed. Furthermore, a control law for slip estimation was developed for slip-affected systems. To obtain the tracking performance, a traditional PID algorithm was also implemented for comparison. Simulations were conducted to tackle trajectory-tracking problems for shipborne climbing manipulation. The results revealed that during a circular trajectory-tracking case with a minimum curvature radius of 0.5 m, precision was maintained below 15 mm. Similarly, during linear tracking in mobile operations, the precision remained below 10 mm. The precision slightly decreased for a circular trajectory with a minimum curvature radius when it was applied for climbing manipulation on 50° IP and 60° IP. However, the precision for a linear trajectory remained around 10 mm. Finally, the proposed approach was deployed for a real shipborne climbing robot, and experiments were performed on uncertain inclined planes, e.g., HP, 50° IP, 60° IP, and VP. All the errors of shipborne climbing-oriented linear trajectory tracking are less than 15 mm, with a minimum RMSE of 6 mm. The algorithm that considers slippage reduces the error caused by slippage by 30% even in the worst-case scenario. As a result, the simple yet stable skid-steering method demonstrates good applicability in the field of wall-climbing manipulation.

## Figures and Tables

**Table 1:** Comparison of the adaptive robust controller with traditional PID control approach.

Index	Criteria	PID Controller	Adaptive Controller
1	Adaptability to variable conditions	Tuned for specific conditions	Adapts dynamically to changes in slip conditions
2	Handling unknown parameters	Relies on fixed parameters	Estimates and adapts to unknown slip parameters
3	Stability guarantee	Lacks robust stability guarantees	Utilizes Lyapunov theory for a mathematically guaranteed stability
4	Improved performance in slip-prone scenarios	Struggles in slip-prone scenarios	Specifically designed for enhanced performance in slip-prone environments
5	Reduced energy consumption	Control efforts may not be optimized	Potential for optimized control actions, leading to reduced energy consumption
6	Control approach	Geometric-based	Model-based

**Table 2:** Configurations of hardware and parameters.

Parameters	Value	Parameters	Value
Processor	Intel i7-9750H 2.6 GHz	Operating system	Win 10, ×64
RAM	32 G	Software tool	MATLAB
Index	Parameters	Value	Unit
1	R, desired radius of the circular trajectory	1.5	[m]
2	W, width of wheelbase	0.22	[m]
3	r, each wheel radius	0.10	[m]
4	b, instantaneous center of rotation (ICR)	0.0107	[m]
5	$K_{p}^{l}$, proportional gain for linear velocity control	60	--
6	$K_{I}^{l}$, integral gain for linear velocity control	10	--
7	$K_{D}^{l}$, derivative gain for linear velocity control	0.01	--
8	$K_{p}^{a}$, proportional gain for angular velocity control	50	--
9	$K_{I}^{a}$, integral gain for angular velocity control	10.0	--
10	$K_{D}^{a}$, derivative gain for angular velocity control	0.01	--

**Table 3:** Simulation parameters for adaptive kinematic control algorithm.

Index	Parameters	Value	Unit
1	R, desired radius of the circular trajectory	1.5	[m]
2	W, width of wheelbase	0.44/2	[m]
3	r, each wheel radius	0.10	[m]
4	b, instantaneous center of rotation (ICR)	0.0107	[m]
5	k1, controller gain to tune the linear velocity	9.0	--
6	k2, controller gain to tune the angular velocity	150.0	--
7	k3, controller gain to tune the angular velocity	0.5	--
8	$s_{r}$, slip control gain for right wheel pair	961.9516	--
9	$s_{l}$, slip control gain for left wheel pair	0.8695	--

**Table 4:** Simulation results.

Orientation	RMSE			
	Angular Velocity rad/s	Linear Velocity m/s,	X-Position m	Y-Position m
Vertical	0.0928	0.0711	0.014	0.013
Horizon	0.0839	0.0654	0.0037	0.0098
45°	0.0695	0.0568	0.0018	0.0015
135°	0.0567	0.0613	0.0015	0.0013

**Table 5:** Geometric parameters and model configuration.

Index	Category	Parameters	Configuration	On-Site Experiment Scene
1	Basic	Size	660 mm × 640 mm × 250 mm	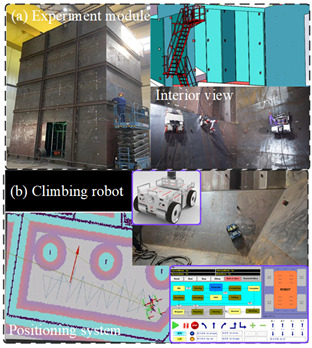
2		Weight	70 Kg	
3		Adsorption	Permanent magnet	
4	Mobility	Maximum velocity	2 m/s	
5		Minimum radius	0.5 m	
6		Power	48 V DC lithium battery	
7	Control system	Controller	Beckhoff c6930	
8		Communication	EtherCAT	
9	Positioning	Controller	Jetson AGX Xavier	
10		Sensor	Livox Mid360	

**Table 6:** The performance of linear trajectory tracking with or without slip consideration.

RMSE of Linear Tracking	*x*				*y*			
	HP	50° IP	60° IP	VP	HP	50° IP	60° IP	VP
Without slip consideration	0.0112	0.0283	0.0525	0.0632	0.0217	0.0247	0.0553	0.0604
Considering slip	0.0087	0.0067	0.0108	0.0073	0.0099	0.0138	0.0072	0.0092
Improvement	77.68%	23.67%	20.57%	11.55%	45.62%	55.87%	13.02%	15.23%

## Data Availability

Data are contained within the article.
